# *Euodia pasteuriana* Methanol Extract Exerts Anti-Inflammatory Effects by Targeting TAK1 in the AP-1 Signaling Pathway

**DOI:** 10.3390/molecules25235760

**Published:** 2020-12-07

**Authors:** Jianmei Zhang, Mi-Yeon Kim, Jae Youl Cho

**Affiliations:** 1 Department of Integrative Biotechnology, and Biomedical Institute for Convergence at SKKU (BICS), Sungkyunkwan University, Suwon 16419, Korea; zhangjianmei1028@163.com; 2 School of Systems Biomedical Science, Soongsil University, Seoul 06978, Korea

**Keywords:** *Euodia pasteuriana*, anti-inflammatory, AP-1, TAK1

## Abstract

*Euodia pasteuriana* A. Chev. ex Guillaumin, also known as *Melicope accedens* (Blume) T.G. Hartley, is a herbal medicinal plant native to Vietnam. Although *Euodia pasteuriana* is used as a traditional medicine to treat a variety of inflammatory diseases, the pharmacological mechanisms related to this plant are unclear. This study aimed to investigate the anti-inflammatory effects of a methanol extract of *Euodia pasteuriana* leaves (Ep-ME) on the production of inflammatory mediators, the mRNA expression of proinflammatory genes, and inflammatory signaling activities in macrophage cell lines. The results showed that Ep-ME strongly suppressed the release of nitric oxide (NO) in RAW264.7 cells induced with lipopolysaccharide (LPS), pam3CysSerLys4 (Pam3CSK), and polyinosinic-polycytidylic acid (poly I:C) without cytotoxicity. A reverse transcription-polymerase chain reaction further confirmed that Ep-ME suppressed the expression of interleukin 6 (IL-6), matrix metalloproteinase-1 (MMP1), matrix metalloproteinase-2 (MMP2), matrix metalloproteinase-3 (MMP3), tumor necrosis factor-α (TNF-α), and matrix metalloproteinase-9 (MMP9) at the transcriptional level and reduced the luciferase activities of activator protein 1 (AP-1) reporter promoters. In addition, immunoblotting analyses of the whole lysate and nuclear fraction, as well as overexpression assays demonstrated that Ep-ME decreased the translocation of c-Jun and suppressed the activation of transforming growth factor beta-activated kinase 1 (TAK1) in the AP-1 signaling pathways. These results imply that Ep-ME could be developed as an anti-inflammatory agent that targets TAK1 in the AP-1 signaling pathway.

## 1. Introduction

Inflammation is a fast, complex biological response in mammals that aids in the elimination of harmful stimuli and the repair of damaged tissue [1]. Immune cells, such as neutrophils, monocytes, and macrophages, can be quickly recruited to sites of injury and inflammation, where they identify foreign invaders and release chemokines, cytokines, and eicosanoids to regulate immunity and restore the body’s physiological balance [2,3].

Among immune cells, macrophages have been most extensively investigated. Numerous studies have attempted to explain the potential molecular mechanism by which various proinflammatory stimuli in macrophages cause an inflammatory response [4,5]. The pathogen-associated molecular pattern (PAMP) is a complex formed through pathogen recognition receptors (PRRs) in cells and the conserved structure of pathogens, which can be recognized by the related receptor and induce the expression of inflammatory factors and cytokines through a signal cascade of stimulation [6,7]. Once macrophages are triggered by PRRs, these molecules induce an inflammatory response.

Toll-like receptors (TLRs) are transmembrane signal transmission receptors discovered in macrophages as one of the most important PRRs. These receptors mediate the secretion of host-related cytokines and the production of natural immune responses via a variety of PAMPs such as lipopolysaccharide (LPS), pam3CysSerLys4 (Pam3CSK), and polyinosinic-polycytidylic acid (poly I:C) [8,9,10]. Different TLRs have different responses to different pathogens. In particular, LPS can activate TLR4 expressed through macrophages and facilitate inflammatory responses by triggering a signaling cascade [11]. 

TLRs form dimers and transform their structure after recognizing the corresponding factors. Simultaneously, they recruit and induce TIR-domain-containing adaptor-inducing interferon-β (TRIF) and TLR adaptor molecule myeloid differentiation primary response 88 (MyD88). TRIF and MyD88 transduce the signal to downstream cascades and ultimately activate inflammatory transcriptional factors such as nuclear factor-κB (NF-κB) and activating protein-1 (AP-1) [12]. With regard to AP-1, the activated signals from TLRs are transmitted by the interleukin-1 receptor-associated kinase (IRAK)/transforming growth factor β-activated kinase 1 (TAK1)/mitogen-activated protein kinase (MAPK) pathway [13,14]. Interestingly, MAPKs can phosphorylate AP-1 subunits such as c-Fos, c-Jun, and ATF [15]. Furthermore, activated macrophages trigger inflammation through the release of inflammatory cytokines and mediators such as nitric oxide (NO), cyclooxygenase (COX)-2, interleukin (IL)-6, and tumor necrosis factor (TNF)-α [16,17,18]. However, prolonged inflammation could lead to severe chronic inflammatory diseases such as rheumatoid arthritis, diabetes, cancer, asthma, and atherosclerosis [19,20]. Natural products with pharmacological activities extracted or isolated from plants produce anti-inflammatory activities by targeting specific signaling cascades [21]. Nevertheless, the anti-inflammatory potential of numerous plants related to ethnic pharmacology remains to be studied.

*Euodia pasteuriana* A. Chev. ex Guillaumin, also known as *Melicope accedens* (Blume) T.G. Hartley, is a species of Euodia, a genus in the family *Rutaceae*, and is mainly distributed in Vietnam. Although *Euodia pasteuriana* is used in traditional medicine to treat a variety of inflammatory diseases, the pharmacological mechanism related to this plant remains unclear. Accordingly, in this study, we investigated the anti-inflammatory effects and molecular mechanisms of a methanol extract of *Euodia pasteuriana* leaves (Ep-ME) using LPS-induced macrophages and determined a pharmacological target of the extract through an overexpression strategy.

## 2. Results

### 2.1. Effects of Ep-ME on Production of NO and Cytotoxicity

**Figure 1:**
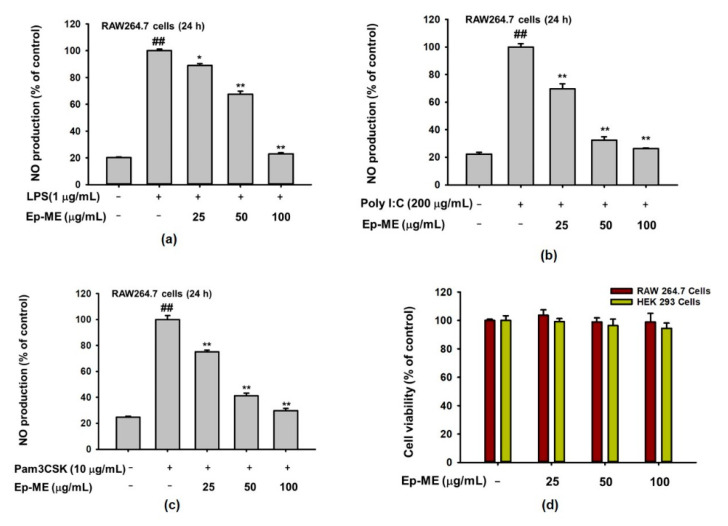
The effects of *Euodia pasteuriana* methanol extract (Ep-ME) on the production of nitric oxide (NO). The level of NO production in the culture supernatant of RAW264.7 cells treated with (**a**) lipopolysaccharide (LPS) (1 μg/mL), (**b**) polyinosinic-polycytidylic acid (poly I:C, 200 μg/mL), or (**c**) Pam3CSK (10 μg/mL) with or without Ep-ME for 24 h. (**d**) The cytotoxic effects of Ep-ME against RAW264.7 cells and HEK293T cells after incubation for 24 h. Data are presented as mean ± SD (*n* = 3). ^##^ *p* < 0.01 vs. untreated control group, * *p* < 0.05, and ** *p* < 0.01 vs. LPS group.

To evaluate the inhibitory activities of Ep-ME on NO production, RAW264.7 cells, a murine macrophage cell line, were pretreated with various concentrations of Ep-ME (0, 25, 50, and 100 μg/mL) for 30 min before adding LPS, Pam3CSK, or Poly I:C, and the NO release levels were examined. As shown in Figure 1a–c, Ep-ME dramatically dose-dependently inhibits the NO production in LPS-, Pam3CSK-, or poly I:C-induced RAW264.7 cells. Specifically, the generation of NO was decreased by up to 22.93%, 26.19%, and 29.70% after treatment with 100 μg/mL of Ep-ME in LPS-, poly I:C-, and Pam3CSK-treated RAW264.7 cells, respectively. In addition, there was no obvious cytotoxicity in RAW264.7 or HEK293T cells after 24 h of incubation with a pharmacologically effective dose of Ep-ME (Figure 1d).

### 2.2. Effect of Ep-ME on Expression of Inflammatory Genes in LPS-Stimulated RAW264.7 Cells

**Figure 2:**
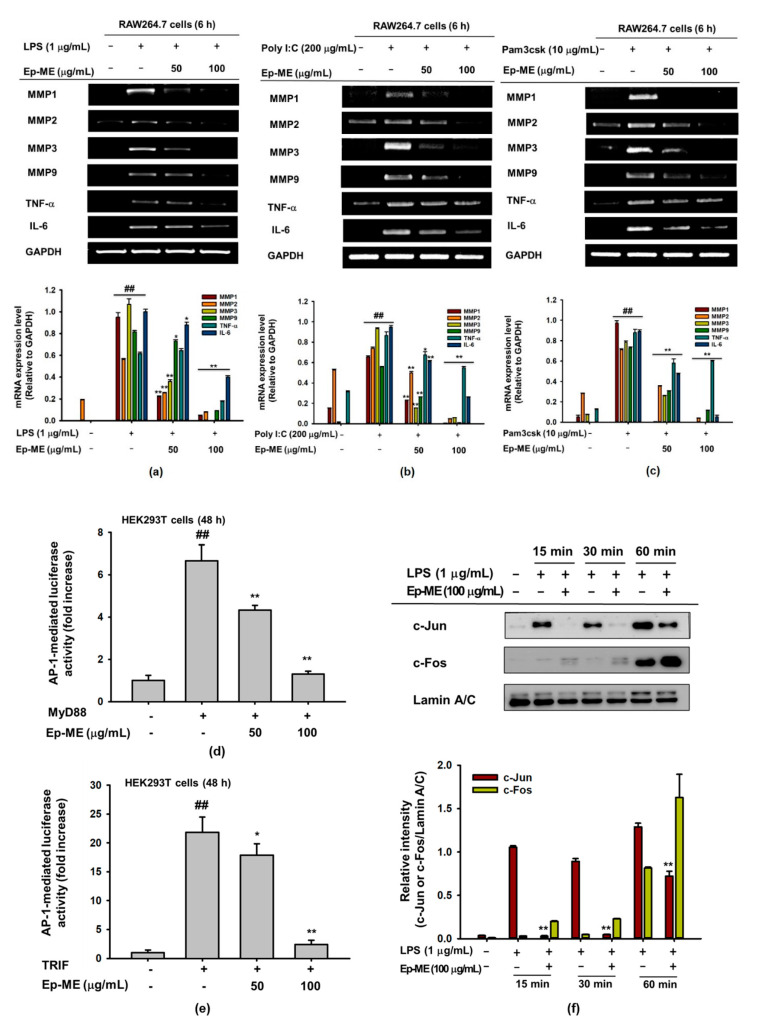
The effects of Ep-ME on the expression of inflammatory genes and transcription factor activation. The mRNA expression levels of MMP1, IL-6, MMP2, TNF-α, MMP3, and MMP9 in (**a**) LPS (1 μg/mL)-, (**b**) poly I:C (200 μg/mL)-, or (**c**) Pam3CSK (10 μg/mL)-induced macrophages treated with Ep-ME were measured by semiquantitative RT-PCR. The relative intensity is quantified through ImageJ. HEK293T cells transfected with (**d**) MyD88 or (**e**) TRIF were transfected with plasmid constructs of AP-1-Luc for 24 h, followed by treatment with Ep-ME for an additional 24 h. (**f**) The nuclear fraction of LPS-stimulated RAW264.7 cells was analyzed using immunoblotting to determine the nuclear translocation levels of the AP-1 subunit (c-Jun and c-Fos) and Lamin A/C. The data presented in (**a**–**f**) are a representative of three independent experiments. ^##^ *p* < 0.01 vs. untreated control group, and * *p* < 0.05 and ** *p* < 0.01 vs. control group.

To demonstrate whether the Ep-ME-mediated suppression of inflammation was involved in the regulation of IL-6, TNF-α, MMP1, MMP2, MMP3, and MMP9 gene expression, RAW264.7 cells were pretreated with Ep-ME for 30 min; induced with LPS, Poly I:C, or Pam3csk for 6 h; and analyzed by RT-PCR. As shown in Figure 2a–c, the mRNA levels of genes related to the AP-1 pathway were upregulated through LPS, poly I:C, or Pam3CSK. In contrast, Ep-ME dramatically dose-dependently downregulated the mRNA expression of MMP1, MMP2, IL-6, TNF-α, MMP3, and MMP9 in LPS-, Pam3CSK-, or poly I:C-stimulated RAW264.7 cells. Because Ep-ME affects the transcriptional levels of inflammatory genes, the effects of Ep-ME on the activation of the inflammatory transcription factor AP-1 were determined by a luciferase reporter gene assay in HEK293T cells. Ep-ME had a dose-dependent inhibitory effect on MyD88- or TRIF-induced AP-1 luciferase gene activities (Figure 2d,e). Furthermore, the nuclear translocation levels of AP-1 subunits (c-Jun and c-Fos) were investigated using nuclear fractionation and immunoblotting analysis. As shown in Figure 2f, the nuclear level of c-Jun was strongly suppressed by Ep-ME at 15, 30, and 60 min after LPS stimulation; however, the level of c-Fos was not inhibited by Ep-ME, which indicated that Ep-ME could diminish the activity of AP-1 by inhibiting the dimerization of AP-1 via the reduction of the nuclear level of c-Jun.

### 2.3. Effect of Ep-ME on Activation of the AP-1 Upstream Signaling Pathway

**Figure 3:**
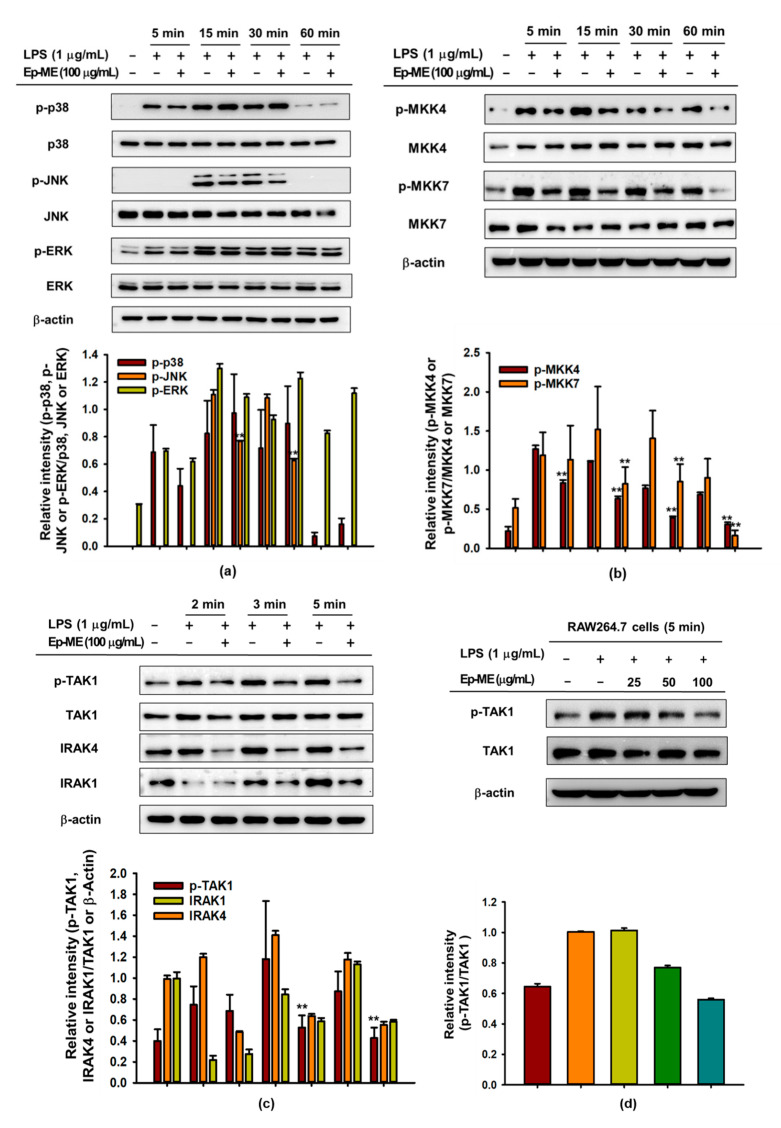
The effects of Ep-ME on the activation of the AP-1 signaling pathway. (**a**–**d**) The levels of the phosphorylated and total forms of JNK, ERK, p38, MKK7, TAK1, MKK4, IRAK1, IRAK4, and β-actin were identified by immunoblotting analyses. The relative intensity is quantified through ImageJ. The data presented in (**a**–**d**) are a representative of three independent experiments. ** *p* < 0.01 vs. control group.

To investigate the modulation of the signaling cascade involved in the AP-1 activity of Ep-ME, the AP-1 upstream signaling cascade was assessed in LPS-induced RAW264.7 cells using immunoblotting analysis. As shown in Figure 3a, the phosphorylation of JNK was inhibited at 15 and 30 min by Ep-ME (100 μg/mL) when compared with cells treated with LPS alone. Similarly, Ep-ME downregulated the phosphorylation of MKK4 and MKK7 (upstream proteins of JNK) at all time points (Figure 3b). Moreover, the expression of phospho-TAK1 (upstream kinase of MAPKKs) was blocked by Ep-ME at earlier time points (2, 3, and 5 min) in LPS-treated RAW264.7 cells in a dose-dependent manner. In contrast, the induction of LPS reduced the protein level of IRAK1 and IRAK4, whereas Ep-ME treatment did not restore the reduced level to a normal state under the same conditions (Figure 3c,d), implying that IRAK-1/4 are not targeted by Ep-ME. This also indicates that TAK1 could be a putative target of Ep-ME in AP-1 inhibitory activities.

### 2.4. Anti-Inflammatory Effects of Ep-ME by Targeting TAK1 Kinase

**Figure 4:**
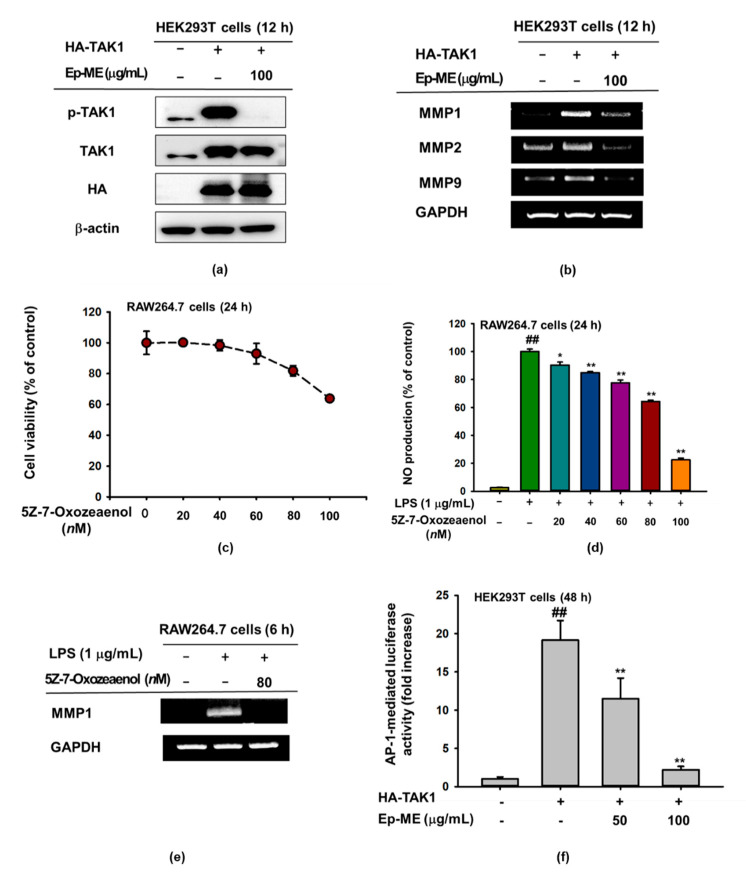
Anti-inflammatory effects of Ep-ME by targeting TAK1 kinases. HEK293T cells were transfected with HA-TAK1, followed by treatment with Ep-ME for an additional 24 h. (**a**) HA, β-actin, and phosphorylated and total forms of TAK1 were examined by immunoblotting. (**b**) The mRNA expression levels of MMP1, GAPDH, MMP2, and MMP9 were assessed by RT-PCR in LPS-induced RAW264.7 cells after treatment with Ep-ME. (**c**) Cytotoxic effects of 5Z-7-oxozeaenol, a TAK1 inhibitor, against RAW264.7 cells after incubation for 24 h. (**d**) NO production was determined through the Griess assay in LPS-induced RAW264.7 cells after treatment with 5Z-7-oxozeaenol. (**e**) The mRNA expression levels of MMP1 and GAPDH were assessed through RT-PCR. (**f**) HEK293T cells transfected with HA-TAK1 plasmid were transfected with AP-1-Luc for 24 h, followed by treatment with Ep-ME for an additional 24 h. The data presented in (**a**,**b**,**e**) are representative of three independent experiments. ^##^ *p* < 0.01 vs. untreated control group, * *p* < 0.05, and ** *p* < 0.01 vs. control group.

To validate the assumption that TAK1 is targeted by Ep-ME, an overexpression strategy was used with an HA-TAK1 plasmid. As expected, Ep-ME significantly inhibited the phosphorylation of TAK1 and dramatically downregulated the mRNA expression of MMP1, MMP2, and MMP9 in HEK293T cells overexpressing TAK1 (Figure 4a,b). Subsequently, we tested the effect of the resorcyclic acid lactone TAK1 inhibitor 5Z-7-oxozeaenol on inflammation [22]. The 5Z-7-Oxozeaenol decreased the NO production of LPS-treated RAW264.7 cells in a dose-dependent manner and had no significant cytotoxicity in the concentration range from 20 nM to 80 nM (Figure 4c,d). Intriguingly, 5Z-7-oxozeaenol dramatically suppressed the mRNA expression of MMP1 at 80 nM in LPS-stimulated RAW264.7 cells (Figure 4e). Finally, the luciferase reporter gene showed that Ep-ME dose-dependently reduced the luciferase activity of TAK1, indicating that it could control TAK1 signaling (Figure 4f). Based on these findings, we confirmed that TAK1 played a crucial role in inflammatory responses.

## 3. Discussion

The aim of this research was to demonstrate the anti-inflammatory effects of *Euodia pasteuriana* and its molecular mechanisms in the AP-1 signaling pathway. A methanol extract of Euodia pasteuriana (Ep-ME) was administered in macrophage models of LPS-induced inflammation, and its pharmacological target in the treatment of inflammation was confirmed. We first investigated whether Ep-ME regulated the production of inflammatory mediators. NO is not only a signaling molecule that plays a crucial role in the pathogenesis of inflammation but also a proinflammation mediator that causes inflammation due to excessive production under abnormal conditions [23]. Therefore, we detected NO secretion in RAW264.7 cells induced with LPS (a TLR4 ligand), pam3CSK (a TLR2 ligand), and poly I:C (a TLR3 ligand). Ep-ME strongly suppressed NO production in LPS-, pam3CSK-, and poly I:C-treated RAW264.7 cells dose-dependently without an obvious cytotoxicity up to 100 μg/mL (Figure 1a–d), indicating that it has the ability to block the production of NO in macrophages.

Proinflammatory cytokines (such as IL-1β, TNF-α, and IL-6) are mainly produced through activated macrophages and participate in the upregulation of inflammatory responses [24]. Recently, several studies have identified that the matrix metalloproteinase (MMP) family regulates the immune response, suppressing inflammation as soluble factors, and that its different members play a critical role in the remission phase of acute inflammation and in regulating inflammatory cytokines, chemokines, and growth factor receptors [25,26,27]. For instance, some MMPs, such as MMP2, MMP3, and MMP9, influence the inflammatory process positively through the activation of pro-IL-1β [28]. Since MMP9 is secreted through inflammatory cells, it could increase arthritis by degrading anti-inflammatory factors, activating inflammatory factors, or promoting the migration of inflammatory cells [29]. Because immune regulation is correlated to post-transcriptional control, we next investigated the mRNA expression of MMP1, MMP2, IL-6, MMP3, TNF-α, and MMP9 genes in LPS-, pam3CSK-, or poly I:C-stimulated RAW264.7 cells. The results of the semiquantitative RT-PCR showed that Ep-ME dramatically downregulated the gene expression of TNF-α, IL-6, MMP1, MMP2, MMP3, and MMP9 in a dose-dependent manner (Figure 2a–c), suggesting that Ep-ME exhibits anti-inflammatory properties.

TLRs are type I transmembrane molecules that play an instructive role in immune responses. TLRs can interact with different combinations of adaptor proteins and transduce downstream signaling via the TRIF-dependent pathway or the MyD88-dependent pathway, before activating the AP-1 and NF-κB signaling pathway to stimulate the production of proinflammatory cytokines [30]. To better understand the anti-inflammatory response of Ep-ME at the molecular level, the AP-1 luciferase reporter gene assay was used in Ep-ME-treated HEK293T cells that were cotransfected with TRIF and MyD88. As shown in Figure 2d,e, the AP-1-driven luciferase activities induced through MyD88 or TRIF transfection were dose-dependently dampened by Ep-ME. The results indicate that Ep-ME exhibits anti-inflammatory effects by targeting the MyD88- or TRIF-mediated AP-1 pathway. Based on the above results, we examined the nuclear translocation level of AP-1 in LPS-induced macrophages using a western blot assay. Surprisingly, the nuclear translocation level of c-Jun of AP-1 subunits was attenuated by Ep-ME treatment at a concentration of 100 μg/mL, although the treatment had no effect on the nuclear translocation level of c-Fos (Figure 2f). Consequently, the present results demonstrate that the suppressive effects of Ep-ME on the mRNA expression of proinflammatory genes and the production of inflammation regulatory molecules were attributed to the inhibition of the nuclear translocation and activation of AP-1.

AP-1, composed of various members such as c-Fos and c-Jun, is a transcription factor that plays a key role in regulating the expression of inflammation-related genes in response to multiple stimuli [31]. Abnormally activated AP-1 is responsible for many inflammatory diseases (including rheumatoid arthritis, sepsis, asthma, and psoriasis) [32]. Hence, the suppression of the AP-1 pathway has become one of the potential methods for the treatment of inflammatory diseases [13,33]. To identify the pharmacological target molecules of Ep-ME in the AP-1 pathway, we analyzed the effect of Ep-ME on intracellular molecules in the AP-1 pathway using western blotting. The AP-1 upstream signaling molecules of JNK, ERK1/2, p38 MAPK, MKK4/7, TAK1, IRAK1, and IRAK4 were assessed in LPS-induced RAW264.7 cells. Ep-ME notably suppressed the level of p-JNK at 15 and 30 min and that of p-MKK4/7 at all times (5–60 min) (Figure 3a,b). As TAK1 is reported to regulate p-MMK4/7 at earlier time points [34], we assessed TAK1, IRAK1, and IRAK4 at 2, 3, and 5 min. As shown in Figure 3c,d, the phosphorylated level of TAK1 was downregulated at 2, 3, and 5 min after LPS induction in RAW264.7 cells, and there was a dose-dependent inhibitory manner at 5 min. However, IRAK1 and IRAK4 were not affected by the Ep-ME treatment. Together, these results demonstrate that TAK1 could be a specific target protein of Ep-ME.

TAK1 is considered a momentous therapeutic target for various types of inflammatory diseases [35,36]. Several studies previously reported that TAK1 could enhance the activities of the downstream molecules MEK1/2, MKK3/6, and MKK4/7 during overexpression [37,38]. These findings imply that TAK1 is immediately regulated through Ep-ME, which was confirmed using the overexpression strategy. The level of p-TAK1 was highly decreased when HEK293T cells were treated with 100 μg/mL (Figure 4a); a similar suppression was obtained at the mRNA level (Figure 4b). Additionally, we further validated the functional effect of the selective TAK1 inhibitor 5Z-7-oxozeaenol, which irreversibly inhibits TAK1 by forming a covalent complex [39,40]. Interestingly, the chemical suppression of TAK1 by 5Z-7-oxozeaenol diminished the activation of AP-1 related to the inhibition of inflammatory responses (Figure 4c–e). The luciferase reporter gene result showed that Ep-ME dose-dependently reduced the luciferase activities of TAK1 (Figure 4f). In addition, these results indicate that Ep-ME can target TAK1 during its anti-inflammatory activity of the AP-1 regulation cascade.

Based on our previous liquid chromatography-mass spectrometry results (LC-MS) (Kim et al., 2020, submitted), Ep-ME contains several biologically active ingredients, such as euxanthone, daidzein, dracorhodin, nevadensin, 5-hydroxyauranetin, 6-hydroxy-7-methoxy-2-(2-phenylethyl) chromone, and so on. It has been reported that euxanthone exerts anti-inflammatory effects by inhibiting the production of TNF-α, IL-1β, and IL-6 in sevoflurane-induced neonatal mice [41]. Peng et al. reported that daidzein dramatically repressed the TNF-α-induced phosphorylation of JNK [42]. Furthermore, daidzein suppressed the production of NO and IL-6 in LPS-treated RAW264.7 cells [43]. Consequently, we speculate that the daidzein and euxanthone in Ep-ME could be responsible for its anti-inflammatory activity.

**Figure 5:**
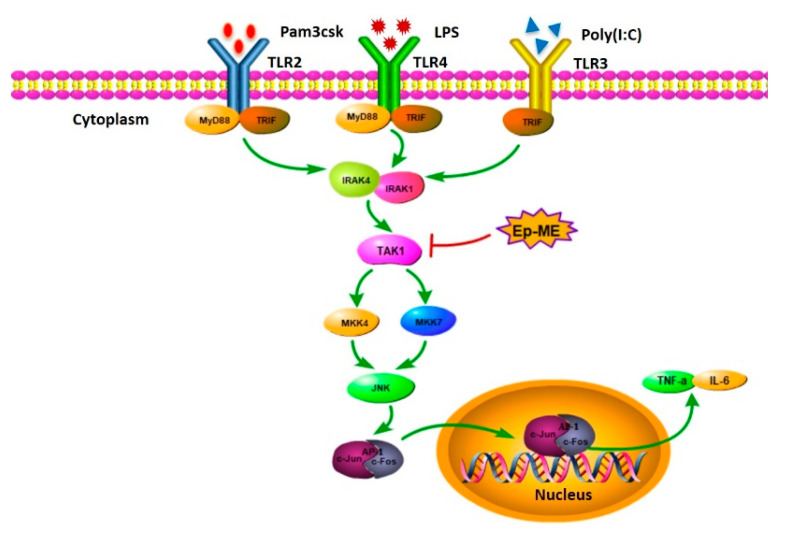
Schematic diagram of the anti-inflammatory activity of Ep-ME when targeting TAK1 in the AP-1 signaling pathway.

In summary, this study demonstrates the potent anti-inflammatory effects of Ep-ME, a methanol extract of *Euodia pasteuriana*, and the abilities of Ep-ME to dampen the production of inflammatory mediators (NO) and the transcription of IL-6, TNF-α, MMP1, MMP2, MMP3, and MMP9, following LPS, pam3CSK, or poly I: C challenges. Furthermore, our findings imply that the mechanism underlying the activities of Ep-ME on inflammatory mediators involves blocking AP-1 nuclear translocation via the downregulation of JNK, MKK4, MKK7, and TAK1 in the LPS-activated AP-1 signaling pathway of macrophages. Hence, the anti-inflammatory activity of Ep-ME could be achieved through the direct suppression of TAK1, an upstream kinase in the AP-1 signaling pathway, as summarized in Figure 5. These data indicate that Ep-ME is a potential herbal medicine candidate for the treatment of inflammatory diseases, and that it could be exploited as a therapeutic agent for the resolution of inflammatory symptoms. This study also provides a better understanding of the inflammatory disease pathway and paves the road for the discovery of new targets for therapeutic applications.

## 4. Materials and Methods

### 4.1. Materials

A methanol extract of the leaves of *Euodia pasteuriana* (Ep-ME) was obtained from the International Biological Material Research Center (Daejeon, Korea). Briefly, dried and refined leaves of Euodia pasteuriana (100 g) were extracted with 1 L of 95% methanol for 2 h, twice. The extract was percolated with filter paper (3 mm; Whatman PLC, Kent, UK), condensed using a Buchi rotary evaporator (Merck, Darmstadt, Germany), and lypophilized using a laboratory freeze dryer (Martin Christ Gefriertrocknungsanlagen GmbH, Harz, Germany) with a 17% yield. Ep-ME was dissolved in 100% dimethylsulfoxide (DMSO) to make stock solution (100 mg/mL) and then diluted with medium. Lipopolysaccharide, 3-(4,5-dimethyl-2-thiazolyl)-2,5-diphenyl-2-H-tetrazolium bromide (MTT), Pam3CSK, dimethylsulfoxide (DMSO), poly I:C sodium salt, 5*Z*-7-oxozeaenol, and polyethylene imidazole (PEI) were obtained from Sigma Chemical Co. (St. Louis, MO, USA). Dulbecco’s modified Eagle’s medium (DMEM), fetal bovine serum (FBS), penicillin-streptomycin solution, phosphate-buffered saline (PBS), and Roswell Park Memorial Institute (RPMI) 1640 were obtained from HyClone (Logan, UT, USA). Trypsin-EDTA (0.25%) was obtained from Corning (Manassas, VA, USA). The antibodies for Lamin A/C, ERK, p-ERK, β-actin, p38, c-Fos, p-p38, MKK7, Jun, p-MKK7, JNK, p-JNK, MKK4, p-MKK4, TAK1, p-TAK1, IRAK4, IRAK1, and HA were purchased from Cell Signaling Technology (Beverly, MA, USA).

### 4.2. Cell Line and Cell Culture

RAW264.7 and HEK293T cells were purchased from the American Type Culture Collection (Rockville, MD, USA). The two cell lines were cultured in RPMI and DMEM containing 10% (or 5%) FBS, 0.1 mg/mL streptomycin, and 100 U/mL penicillin at 37 °C under 5% CO_2_.

### 4.3. Determination of NO Production

RAW264.7 cells were seeded in 96-well plates at a density of 1 × 10^5^ cells per well and cultured overnight. The cells were pretreated with Ep-ME (0, 25, 50, and 100 μg/mL) for 30 min and incubated with LPS (1 μg/mL), poly I:C (200 μg/mL), and Pam3CSK (10 μg/mL) for 24 h. The NO production was measured by Griess reaction, as reported previously [44].

### 4.4. In Vitro Cell Viability Assay

The effects of Ep-ME on the cytotoxicity were assessed through a conventional MTT assay. Briefly, RAW264.7 and HEK293T cells were incubated for 16–20 h and then treated with Ep-ME at concentrations of 0, 25, 50, and 100 μg/mL for 24 h. Subsequently, 10 μL of MTT solution was added to each well and incubated for an additional 3 h, and the purple formazan crystals were solubilized by adding 100 μL of 15% sodium dodecyl sulfate. Finally, the absorbance was measured at 570 nm by a Synergy HT Multi-Mode (Winooski, VT, USA).

### 4.5. mRNA Expression Analysis Using Reverse Transcription-Polymerase Chain Reaction (RT-PCR)

To estimate cytokine mRNA expression levels, such as MMP1, MMP3, IL-6, MMP2, TNF-α, and MMP9, the total RNA was extracted with TRIzol reagent, according to the manufacturer’s instructions, from RAW264.7 cells that had been pretreated with Ep-ME at different concentrations for 30 min before being stimulated with LPS, Pam3CSK, or poly I:C for 6 h. RT-PCR reactions were performed as described in our previous study [45]. The primers used in this experiment were from Bioneer (Seoul, Korea) and are listed in Table 1.

### 4.6. Luciferase Reporter Gene Activity Assay

HEK293T cells (1 × 10^6^ cells/well) were transfected with 1 µg/mL of plasmid including β-gal and AP-1-Luc with or without inducing molecules (MyD88 and TRIF) by a polyethyleneimine (PEI) assay in 24-well plates for 24 h. Then, the cells were treated with Ep-ME (0, 50, and 100 μg/mL) for an additional 24 h. Subsequently, luciferase activities were detected through the luciferase assay system (Promega, WI, USA). To assess the anti-inflammatory activity of Ep-ME on the overexpression of a specific molecule, HEK293T cells were transfected with HA-TAK1 plasmids diluted in Opti-MEM for 24 h.

### 4.7. Preparation of Nuclear Extracts and Whole-Cell Lysates

RAW264.7 or HEK 293T cells were seeded in 60 × 60 mm plates (5 × 10^6^ cells/well) and incubated overnight. Following treatment with Ep-ME (100 µg/mL), the cells were collected in PBS. Whole-cell lysates were extracted by lysing with ice-cold lysis buffer (2 mM ethylenediaminetetraacetic acid (EDTA), 2 mM ethylene glycol tetraacetic acid, 20 mM Tris-HCl, 50 mM glycerol phosphate, 2 µg/mL aprotinin, 1 mM 1,4-dithiothreitol (DTT), 50 µM phenylmethylsulfonyl fluoride (PMSF), 1 mM benzamide, 1 µg/mL pepstatin A, 10% glycerol, 20 mM sodium fluoride, 1.6 mM pervanadate, 2% Triton X-100, 2 µg/mL leupeptin, and 0.1 mM of sodium vanadate) and were centrifuged at 12,000 rpm for 10 min at 4 °C. Nuclear extracts were acquired with lysis buffer A (0.5% Nonidet P-40, 10 mM HEPES, 2 mM magnesium chloride, 1 mM DTT, 2 µg/mL aprotinin, 10 mM potassium chloride (KCl), 0.1 µM PMSF, 2 µg/mL leupeptin, 0.1 mM EDTA) and sonicated to lyse the cells. The lysates were centrifuged at 12,000 rpm for 1 min. The pellet was suspended in extraction buffer (0.1 mM EDTA, 50 mM KCl, 0.1 mM PMSF, 10% glycerol, 1 mM DTT, and 300 mM sodium chloride) and incubated on ice for 25 min. Finally, the supernatant was collected as a nuclear extract by centrifugation for 10 min at 12,000 rpm.

### 4.8. Western Blot Analysis

The protein concentrations of nuclear or whole-cell lysates were quantified with the Bradford assay with BSA as the standard. Proteins were size-dependently resolved through 8–12% SDS-polyacrylamide gel electrophoresis and then transferred to a polyvinylidene difluoride (PVDF) membrane. After blocking PVDF membranes with 3% BSA for 1 h, the membranes were incubated overnight with primary antibody at 4 °C, washed three times with Tris-buffered saline with Tween-20 (TBST), and incubated for 1 h with a secondary antibody. The protein bands were detected using an ECL western blotting kit and photographed by a Tanon-5200 multi-imaging system.

### 4.9. Statistical Analysis

Data are presented as the mean ± standard deviation (SD). The significance was analyzed between the control and treatment groups using Student’s *t*-test. *p* values under 0.05 or 0.01 were considered significant.

## Figures and Tables

**Table 1:** The primer sequences for the RT-PCR analysis.

Gene Name	Direction	Sequences (5′ to 3′)
MMP1 (Mouse)	Forward	ACAACGGAGACCGGCAAAAT
	Reverse	GCTGGAAAGTGTGAGCAAGC
MMP2 (Mouse)	Forward	GCCCCCATGAAGCCTTGTTT
	Reverse	GTCAGTATCAGCATCGGGGG
MMP3 (Mouse)	Forward	ACTCCCTGGGACTCTACCAC
	Reverse	TTCTTCACGGTTGCAGGGAG
MMP9 (Mouse)	Forward	TCTTCCCCAAAGACCTGAAA
	Reverse	TGATGTTATGATGGTCCCAC
TNF-α (Mouse)	Forward	TAGCCCACGTCGTAGCAAAC
	Reverse	ACCCTGAGCCATAATCCCCT
IL-6 (Mouse)	Forward	GCCTTCTTGGGACTGATGCT
	Reverse	TGGAAATTGGGGTAGGAAGGAC
GAPDH (Mouse)	Forward	ACCACAGTCCATGCCATCAC
	Reverse	CCACCACCCTGTTGCTGTAG
MMP1 (Human)	Forward	CACAGCTTCCCAGCGACTC
	Reverse	GTCCCGATGATCTCCCCTGA
MMP2 (Human)	Forward	CCCACTGAGGAGTCCAACAT
	Reverse	CATTTACACGTCTGCGGATCT
MMP3 (Human)	Forward	ATCCTACTGTTGCTGTGCGT
	Reverse	CATCACCTCCAGAGTGTCGG
GAPDH (Human)	Forward	GGTCACCAGGGCTGCTTTTA
	Reverse	GATGGCATGGACTGTGGTCA

## Abbreviations

Ep-ME: *Euodia pasteuriana* methanol extract; NO, nitric oxide; PAMP, pathogen-associated molecular pattern; PRR, pathogen recognition receptor; LPS, lipopolysaccharide; MMP1, matrix metalloproteinase-1; Pam3CSK4, Pam3-Cys-Ser-Lys4; MMP2, matrix metalloproteinase-2; Poly I:C, polyinosinic-polycytidylic acid; MMP3, matrix metalloproteinase-3; TNF-α, tumor necrosis factor α; TLR, Toll-like receptor; NF-κB, nuclear factor-κB; MMP9, matrix metalloproteinase-9; MTT, (3-4,5-dimethylthiazol-2-yl)-2,5-diphenyltetrazolium bromide; AP-1, activator protein 1; IL-6, interleukin 6; MyD88, myeloid differentiation primary response 88; DMSO, dimethyl sulfoxide; FBS, fetal bovine serum; RT-PCR, reverse transcriptase-polymerase chain reaction; TRIF, TIR domain-containing adaptor including interferon-β; TAK1, transforming growth factor beta-activated kinase 1; MAPK, mitogen-activated protein kinase; GAPDH, glyceraldehyde-3-phophate dehydrogenase.
